# Sorbitol production from mixtures of molasses and sugarcane bagasse hydrolysate using the thermally adapted *Zymomonas mobilis* ZM AD41

**DOI:** 10.1038/s41598-024-56307-8

**Published:** 2024-03-06

**Authors:** Yupaporn Phannarangsee, Bunyapon Jiawkhangphlu, Sudarat Thanonkeo, Preekamol Klanrit, Mamoru Yamada, Pornthap Thanonkeo

**Affiliations:** 1 Department of Biotechnology, Faculty of Technology, Khon Kaen University, Khon Kaen, 40002 Thailand; 2 Walai Rukhavej Botanical Research Institute (WRBRI), Mahasarakham University, Maha Sarakham, 44150 Thailand; 3 Fermentation Research Center for Value Added Agricultural Products (FerVAAP), Khon Kaen University, Khon Kaen, 40002 Thailand; 4 Department of Biological Chemistry, Faculty of Agriculture, Yamaguchi University, Yamaguchi, 753-8515 Japan; 5 Research Center for Thermotolerant Microbial Resources, Yamaguchi University, Yamaguchi, 753-8515 Japan

**Keywords:** High-temperature fermentation, Sorbitol production, Sugarcane bagasse, Sugarcane molasses, Thermotolerant microbes, *Zymomonas mobilis*, Biotechnology, Microbiology

## Abstract

Byproducts from the sugarcane manufacturing process, specifically sugarcane molasses (SM) and sugarcane bagasse (SB), can be used as alternative raw materials for sorbitol production via the biological fermentation process. This study investigated the production of sorbitol from SM and sugarcane bagasse hydrolysate (SBH) using a thermally adapted *Zymomonas mobilis* ZM AD41. Various combinations of SM and SBH on sorbitol production using batch fermentation process were tested. The results revealed that SM alone (FM1) or a mixture of SM and SBH at a ratio of 3:1 (FM2) based on the sugar mass in the raw material proved to be the best condition for sorbitol production by ZM AD41 at 37 °C. Further optimization conditions for sorbitol production revealed that a sugar concentration of 200 g/L and a CaCl_2_ concentration of 5.0 g/L yielded the highest sorbitol content. The maximum sorbitol concentrations produced by ZM AD41 in the fermentation medium containing SM (FM1) or a mixture of SM and SBH (FM2) were 31.23 and 30.45 g/L, respectively, comparable to those reported in the literature using sucrose or a mixture of sucrose and maltose as feedstock. These results suggested that SBH could be used as an alternative feedstock to supplement or blend with SM for sustainable sorbitol production. In addition, the fermentation conditions established in this study could also be applied to large-scale sorbitol production. Moreover, the thermally adapted *Z. mobilis* ZM AD41 is also a promising sorbitol-producing bacterium for large-scale production at a relatively high fermentation temperature using agricultural byproducts, specifically SM and SB, as feedstock, which could reduce the operating cost due to minimizing the energy required for the cooling system.

## Introduction

The natural sugar alcohol sorbitol or d-glucitol (C_6_H_14_O_6_) is one of the top 12 high-value-added products from biomass and can be applied in various industries, such as food, pharmaceuticals, cosmetics, and textiles^1–4^. In the food industry, sorbitol can be used not only as a sweetener but also as a texturizer, moisturizer, and softener. Sorbitol has been used in dietetic foods for diabetic patients since its metabolic pathway is noninsulin-dependent^5^. Due to high demand, the global production of sorbitol is estimated to be approximately 2 million tons per year, and its market is continuously increasing^6^.

Renewable biomass has emerged as a high-potential feedstock for producing bioenergies, biopolymers, and several other platform chemicals and added-value products through the biorefinery concept. In the past decade, sorbitol has been primarily produced via chemical processes, mainly through catalytic hydrogenation of glucose^5,7,8^ or via biological fermentation of sucrose or a mixture of sucrose and maltose or glucose and fructose^9–12^. Due to the negative environmental impact of the chemical process and the high cost of the sugar materials used in biological fermentation, the biological production of sorbitol using low-cost and abundant renewable biomass, specifically byproducts from the agricultural industry, has received significant attention.

Sugarcane is one of the most significant agricultural and economic crops in Thailand and other countries, such as Brazil, India, and China. Based on global sugarcane production in 2019, Thailand is the fourth largest producer after Brazil, India, and China, producing approximately 131 million tonnes (Mt) annually, accounting for 6% of the world production^13^. Fifty-six sugar factories were operated in Thailand, generating a total sugar of 1.46 Mt. In addition to sugar as the main product, the sugar manufacturing process also generated other byproducts, including approximately 5.88 Mt of sugarcane molasses (SM) and 48.4 Mt of sugarcane bagasse (SB) accounts for 44% and 37% of sugarcane raw materials, respectively^14^. Recently, SM has been mainly used as a feedstock for bioethanol production, whereas SB is used as fuel to generate heat in boiling systems and thermal power plants. Previous studies demonstrated that SM contained high amounts of soluble carbohydrates, specifically sugars, such as sucrose, glucose, and fructose, while SB contained primarily insoluble carbohydrates, mainly cellulose and hemicellulose, which can be converted into fermentable sugars either by chemical, physical, or biological processes^15,16^. Due to their high content of polysaccharides and being renewable resources, inexpensive, and readily available, these byproducts show promising potential as feedstocks for sorbitol production via a biological fermentation process.

*Zymomonas mobilis*, a Gram-negative facultative anaerobic bacterium, is among the most promising sorbitol-producing microorganisms since it possesses several ideal industrial biocatalyst properties, such as low biomass yield, low oxygen requirements, high osmolarity tolerance, high ethanol tolerance, high sugar uptake and conversion rate, and high product formation rate^17^. Several strains of *Z. mobilis* have been investigated for their sorbitol production efficiency, such as *Z. mobilis* 113S^18^, CP4^9^, ZM4 (ATCC 31,821)^19^, ATCC 29,191^10,11,20^, pHW20a-*gfo*^12^, and TISTR548^16^. Most of these strains, except strain TISTR548, could grow and produce high sorbitol concentrations at a normal growth temperature (30 °C). Based on the literature review, little information is available on sorbitol production employing a relatively high-temperature fermentation (HTF) platform using thermotolerant *Z. mobilis*, which provides several advantages, such as high substrate uptake and conversion rate, high fermentation yield, and low operating cost due to a reduction in the energy input of the cooling system. Furthermore, HTF can minimize the risk of contamination of undesired microorganisms during fermentation^21,22^.

Several strains of *Z. mobilis* have been developed in our laboratory, and among these strains, the thermally adapted strain ZM AD41 exhibited high growth performance under various conditions, including high temperatures, high sugar, and hydrogen peroxide (H_2_O_2_) concentrations. Interestingly, it can grow at a high temperature of 41 °C and tolerate a relatively high concentration of acetic acid, one of the lignocellulosic inhibitors that negatively affect the growth of several microbes^21^. The sorbitol production from this thermally adapted strain at HTF condition has never been documented. Therefore, this study aims to investigate the sorbitol production potential of *Z. mobilis* ZM AD41 using byproducts from the sugarcane manufacturing process, including SM and SB as feedstocks. The effect of sugar concentrations and metal ions on sorbitol production by ZM AD41 was also determined.

## Materials and methods

### Chemicals

Yeast extract and peptone (bacteriological grade) were purchased from TM Media (Titan Biotech Ltd., India). Glucose (analytical grade) was purchased from KemAusTM, Australia. Sorbitol and ethanol (HPLC grade) were procured from Sigma − Aldrich (St. Louis, MO, USA). Agar (bacteriological grade) and other chemicals, such as zinc sulfate (ZnSO_4_.7H_2_O), calcium chloride (CaCl_2_), iron sulfate (FeSO_4_.7H_2_O), magnesium sulfate (MgSO_4_.7H_2_O), manganese sulfate (MnSO_4_.H_2_O), and copper sulfate (CuSO_4_.5H_2_O) (analytical grade) were obtained from a local supplier (CLS supply and Services, Ltd. Part., Khon Kaen, Thailand).

### *Z. mobilis* strain and culture medium

A thermally adapted *Z. mobilis* strain ZM AD41 was used in this study. This bacterial strain was developed in our laboratory by Samappito et al.^21^ using adaptive laboratory evolution (ALE) under long-term repeated cultivation by gradually increasing the incubation temperature from 38 to 41 °C for 230 cycles. It was maintained at 4 °C in yeast extract peptone glucose (YPG) agar medium comprising yeast extract (3.0 g/L), peptone (5.0 g/L), glucose (20.0 g/L), and agar (15.0 g/L) with subculturing every month. The bacterial inoculum was prepared using the method described by Chamnipa et al.^16^.

### Plant materials and preparation of sugarcane bagasse hydrolysate (SBH)

SM and SB used as feedstocks for sorbitol production in this study were kindly provided by the sugar factory in Khon Kaen province, Thailand, with the permission of the chief executive officer. SM was kept at the Department of Biotechnology, Faculty of Technology, Khon Kaen University, in a storage container at − 20 °C with the code number KKUDB-SM-2022-01, while SB was kept in a plastic bag at room temperature with the code number KKUDB-SB-2022-01. All procedures for plant preparation followed the relevant guidelines in the methods section.

SM was filtered through four layers of cheesecloth and centrifuged at 8000 rpm for 10 min before use. The SB was sun-dried for 3 days, followed by oven-drying at 60 °C for 12 h. The resulting dried SB was ground using a feed grinding machine, and a 1.0 − 2.0 mm particle size was collected after sieving. The SB hydrolysate (SBH) was prepared using the procedure described by Chamnipa et al.^23^ using dilute acid pretreatment and an enzymatic hydrolysis process. Sulfuric acid at 2% (v/v) was used for dilute acid pretreatment, while Cellic® Ctec2 (Novozymes, Denmark) (193 filter paper units (FPU)/mL) at a concentration of 50 FPU per gram dry solid (SD) was applied for enzymatic hydrolysis of SB. The obtained supernatant, after centrifugation at 8000 rpm for 10 min, was used to make a fermentation medium for sorbitol production by mixing with SM. The total sugars in the SBH were concentrated to 250 g/L by vacuum evaporation before being mixed with SM.

The chemical compositions of SM and SBH, such as sugars (sucrose, glucose, fructose, maltose, xylose, and arabinose), lignocellulosic inhibitors (acetic acid, furfural, 5-hydroxymethyl furfural), vitamins (thiamine (B1), riboflavin (B2), nicotinamide (B3), pantothenic acid (B5), pyridoxine (B6), folate (B9), and cobalamin (B12)), and trace elements (nitrogen (N), phosphorus (P), potassium (K), magnesium (Mg), calcium (Ca), sulfur (S), iron (Fe), manganese (Mn), zinc (Zn), copper (Cu), sodium (Na), chloride (Cl), and boron (B)), were determined. Sugars, lignocellulosic inhibitors, and vitamins were evaluated using high-performance liquid chromatography (HPLC), while macro- and microelements were analyzed using methods 985.01 and 981.10 of the AOAC^24,25^. All these analyses were performed by the Central Laboratory (Thailand) Co., Ltd., Khon Kaen, Thailand.

### Sorbitol production from a mixture of SM and SBH by *Z. mobilis* ZM AD41

The fermentation medium for sorbitol production (in 1 L) was prepared by mixing SM and SBH at various ratios based on the sugar mass in the raw materials: 1:0 (FM1), 3:1 (FM2), 1:1 (FM3), 1:3 (FM4), and 0:1 (FM5). After mixing, the final sugar concentrations in the fermentation media were adjusted to 150 g/L. The initial pH of all the fermentation media was adjusted to 5.5 using 1 N HCl/NaOH, and all the media were supplemented with yeast extract (3.0 g/L) and peptone (5.0 g/L)^16^.

A 10% (v/v) solution of ZM AD41 active cells was transferred into a 250-mL Erlenmeyer flask filled with 100 mL fermentation medium for sorbitol production. The fermentation was carried out under semi-anaerobic conditions by closing the flasks with cotton plugs and aluminum foil and statically incubating them at 37 °C. The fermentation broths were randomly withdrawn at certain time intervals. The sorbitol and ethanol contents produced by ZM AD41 using different fermentation media were measured by HPLC and gas chromatography (GC), respectively. The fermentation medium with the highest sorbitol content was selected for further study.

### Effect of sugar concentrations on sorbitol production by *Z. mobilis* ZM AD41

The effect of sugar concentrations on sorbitol production by ZM AD41 was evaluated by varying the sugar content in the fermentation medium from 150 to 300 g/L. Fermentation was started by transferring 10% (v/v) bacterial inoculum into a 250-mL Erlenmeyer flask filled with 100 mL of fermentation medium. The flasks were closed with cotton plugs and aluminum foil and then statically incubated at 37 °C. During sorbitol fermentation, culture broths were sampled at specific times, and the sorbitol and ethanol concentrations were measured using HPLC and GC, respectively.

### Effect of metal ions on sorbitol production by *Z. mobilis* ZM AD41

The effect of various divalent metal ions, including ZnSO_4_.7H_2_O, CaCl_2_, FeSO_4_.7H_2_O, MgSO_4_.7H_2_O, and MnSO_4_.H_2_O, and CuSO_4_.5H_2_O on sorbitol production by thermally adapted strain ZM AD41 was determined. The fermentation medium was supplemented with metal ions at different concentrations, which were selected based on the studies of Chang et al.^26^ and Liu et al.^12^. Fermentation was initiated by inoculating 10% (v/v) bacterial starter culture into a 250-mL Erlenmeyer flask filled with 100 mL fermentation medium. After closing the flasks with cotton plugs and aluminum foil, they were statically incubated at 37 °C. The fermentation broths were randomly collected during fermentation, and the sorbitol and ethanol contents were analyzed using HPLC and GC, respectively.

The up-scale production of sorbitol in a 2-L Erlenmeyer flask by ZM AD41 was also determined. The fermentation media with appropriate sugar concentration and metal ions that gave the highest sorbitol content were selected for this experiment. Fermentation was carried out under semi-anaerobic conditions by closing the flasks with cotton plugs and aluminum foil and statically incubated at 37 °C. The chemical changes during fermentation, such as sorbitol, ethanol, and total sugar content, were monitored.

### Morphology analysis of bacterial cells during sorbitol fermentation

The morphology of *Z. mobilis* ZM AD41 was monitored during sorbitol fermentation in different fermentation media. Cells were randomly collected, centrifuged at 10,000 rpm and 4 °C for 5 min, and washed twice with sterile distilled water. After suspending in 0.85% sodium chloride, the resulting cells were imaged using a microscope (Primo Star, Carl Zeiss, Carl Zeiss Co., Ltd., Bangkok, Thailand).

### Analytical methods

The total sugar concentration in the fermentation medium was determined using the phenol‒sulfuric acid method^27^. The sorbitol concentration was analyzed by HPLC (Waters 2414, Waters Corp., Massachusetts, USA) equipped with a refractive index detector (RID) and an Aminex HPX-87 H column using the measurement procedure of Chamnipa et al.^16^. The ethanol concentration was determined using GC (Shimadzu GC-14B, Japan) equipped with a polyethylene glycol (PEG-20 M)-packed column and a flame ionization detector (FID) using the measurement procedure of Chamnipa et al.^16^. Isopropanol was used as an internal standard. All of the experiments were carried out in triplicate, and the data are presented as the means ± standard deviations (SDs). The SPSS program for Windows (IBM SPSS Statistics 28, IBM Corporation, Armonk, NY, USA) was used for statistical analysis, and the mean difference between each treatment was determined based on Duncan’s multiple range test (DMRT) at a probability level of 5%.

## Results and discussion

### Chemical components of SM and SBH

SM comprised 560.28 g/L total sugars, in which sucrose was the primary sugar detected in this feedstock, with a concentration of 325.68 g/L, followed by fructose (143.06 g/L) and glucose (91.54 g/L) (Table 1). Other sugars, including maltose and xylose, were not detected in the raw material. The sugar contents in the SM used in this study differed from those reported in previous works. For instance, Yatim et al.^28^ reported a total sugar content of 509 g/L, composed of 410 g/L sucrose, 67 g/L glucose, and 32 g/L fructose. Another study by Thanapornsin et al.^29^ reported that SM contains 673.26 g/L total sugars comprising 445.60 g/L sucrose, 128.20 g/L glucose, and 99.46 g/L fructose. The difference in the sugar content of SM from different works may be due to the difference in plant variety, planting area, growth environmental conditions, and the sugar manufacturing process.

**Table 1:** Sugars, trace elements, and vitamins in sugarcane molasses (SM) used in this study and those reported in the literature.

Chemical constituents	SM^1^	Thanapornsin et al.^29^	Jamir et al.^30^
Sugars (g/L)			
Sucrose	325.68	445.60	290 − 400
Fructose	143.06	99.46	NA
Glucose	91.54	128.20	40 − 140
Trace elements (g/L)			
N	4.51	NA	NA
P	1.04	0.48	NA
K	17.20	28.13	40 − 508
Mg	4.04	4.50	10 − 140
Ca	3.26	8.11	8 − 150
S	0.61	5.21	NA
Fe	2.01	0.15	NA
Mn	0.90	0.08	NA
Zn	0.33	0.03	NA
Cu	0.12	0.0003	NA
B	0.44	0.05	NA
Cl	1.84	NA	NA
Vitamins (mg/L)			
Nicotinamide (B3)	6.40	NA	NA
Pantothenic acid (B5)	3.13	NA	NA
Pyridoxine (B6)	2.02	NA	NA

*NA* not available.

^1^SM used in this study.

SM also contains various macro- and microelements, and some of them are essential for microbial growth and development, as well as enzyme activity involved in metabolic pathways for sorbitol production. As the results showed in this study, K was the major macroelement found in SM, with a concentration of 17.20 g/L, followed by N (4.51 g/L), Mg (4.04 g/L), Ca (3.26 g/L), P (1.04 g/L), and S (0.61 g/L). Regarding microelements in SM, Fe was the main element found in this feedstock, with a concentration of 2.01 g/L, followed by Cl (1.84 g/L), Mn (0.90 g/L), B (0.44 g/L), Zn (0.33 g/L), and Cu (0.12 g/L) (Table 1). The contents of almost all macroelements detected in the current study, except P, were remarkably lower than those reported by Thanapornsin et al.^29^ and Jamir et al.^30^. On the contrary, the contents of microelements were significantly higher than those reported by Thanapornsin et al.^29^. Although SM used in this study and that used in a study of Thanapornsin et al.^29^ was obtained from the sugar factory in Khon Kaen Province, it may come from a different batch of production making the difference in the chemical components of raw material. In addition to sugars and macro- and microelements, SM also contains vitamins, including B3 (6.40 mg/L), B5 (3.13 mg/L), and B6 (2.02 mg/L). Notably, other vitamins, such as B1, B2, B9, and B12, were not detected in the SM used in this study. As previously reported, several microorganisms require vitamins for their growth and metabolism, either as coenzymes or as important metabolic precursors^31,32^.

The SBH used in the current study was the same batch as that used in a study by Chamnipa et al.^16^. As previously reported, it comprised 85.4 g/L glucose and 24.2 g/L xylose (Table 2). No arabinose sugar was detected in the raw material. The glucose and xylose contents of the SBH reported here were significantly higher than those in the studies of Cheng et al.^33^ and Silva et al.^34^, which might be associated with the difference in plant variety, planting area, harvesting and manufacturing process, and preparation method of SBH production.

**Table 2:** Sugars, trace elements, and acetic acid in sugarcane bagasse hydrolysate (SBH) used in this study and those reported in the literature.

Chemical constituents	SBH^1^	Cheng et al.^33^	Silva et al.^34^
Sugars (g/L)			
Glucose	85.4	9.3	2.2
Xylose	24.2	45.0	40.0
Acetic acid (mM)	21.2	139.9	56.6
Trace elements (mg/L)			
N	804.2	NA	NA
P	27.0	NA	NA
K	46.0	NA	NA
Mg	13.0	NA	NA
Ca	88.0	NA	NA
S	24.0	NA	NA
Fe	4.5	NA	NA
Mn	0.2	NA	NA
Zn	4.0	NA	NA
Cu	0.4	NA	NA
Na	1710.0	NA	NA

*NA* not available.

^1^SBH used in this study^16^.

Several macroelements, such as N (804.2 mg/L), P (27.0 mg/L), K (46.0 mg/L), Ca (88.0 mg/L), Mg (13.0 mg/L), and S (24.0 mg/L), and microelements, such as Na (1,710.0 mg/L), Fe (4.5 mg/L), Zn (4.0 mg/L), Cu (0.4 mg/L), and Mn (0.2 mg/L), were detected in the SBH (Table 2). Among the trace elements, N and Na were the major macro- and microelements in the SBH, respectively. In addition, a lignocellulosic inhibitor, specifically acetic acid, which is generated by the hydrolysis of the acetyl group of hemicellulose during the acid pretreatment process^33–36^, was also detected. This aliphatic and weak acid has been shown to inhibit the growth of microbial cells, particularly at a high concentration level^21^. The concentration of acetic acid detected in the lignocellulosic hydrolysate varied depending on the type of feedstock, pretreatment conditions, and pretreatment method. In this study, the acetic acid concentration in the SBH was 21.2 mM, remarkably lower than those studies by Cheng et al.^33^ and Silva et al.^34^. Notably, other lignocellulosic inhibitors, such as furfural and 5-hydroxymethyl furfural, were not detected in the raw material.

### Sorbitol production from a mixture of SM and SBH by *Z. mobilis* ZM AD41

**Figure 1:**
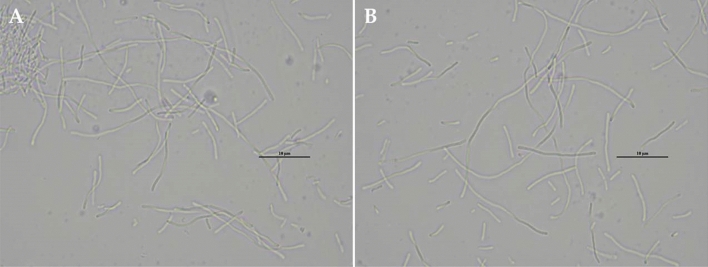
Cell morphology of *Z. mobilis* ZM AD41 grown in fermentation medium containing SBH (FM5 medium) after 24 h (**A**) and 48 h (**B**) of cultivation at 37 °C. Image with 1000 × magnification.

Although a thermally adapted *Z. mobilis* ZM AD41 could grow at a temperature of up to 41 °C^21^, its growth and sorbitol production at 41 °C using SM or SBH as feedstock was deficient. Therefore, in this study, the sorbitol production potential of a thermally adapted strain ZM AD41 was tested at 37 °C using a mixture of SM and SBH as feedstock at various ratios. As shown in Table 3, ZM AD41 produced the highest sorbitol concentration in the fermentation medium composed of SM alone (FM1 medium), yielding the maximum sorbitol content of 9.80 g/L at 24 h and 19.24 g/L at 48 h after fermentation. Longer fermentation times led to a reduction in sorbitol production. Interestingly, a combination of SM and SBH at a ratio of 3:1 (FM2 medium) also gave a relatively high sorbitol content comparable to that obtained from the FM1 medium. The maximum sorbitol concentrations produced in the FM2 medium were 9.05 and 18.94 g/L at 24 and 48 h after fermentation, respectively. This finding suggested that a thermally adapted *Z. mobilis* ZM AD41 can utilize carbon sources and nutrients in SBH for its growth and sorbitol production. Notably, higher concentrations of SBH in the fermentation medium, i.e., SM and SBH ratios of 1:1 (FM3 medium), 1:3 (FM4 medium), and 0:1 (FM5 medium), resulted in a reduction in sorbitol production. Specifically, the fermentation medium containing SBH alone (FM5 medium) provided the lowest sorbitol concentration, approximately 22-fold and 34-fold lower than those in the FM1 medium at 24 h and 48 h after fermentation, respectively. These results suggested that SBH alone may not be suitable for sorbitol production by ZM AD41. One possibility is that the SBH contained a high concentration of Na, particularly in the concentrated SBH after vacuum evaporation. It has been previously reported that a high concentration of Na causes a reorganization of bacterial cell wall structure and inhibits bacterial cell growth by preventing sugar uptake, especially in glucose medium^19^. As observed in this study, cells of ZM AD41 become elongated when grown in SBH (Fig. 1), similar to those studies of Vriesekoop et al.^19^, Fuchino and Bruheim^37^, and Chamnipa et al.^16^.

**Table 3:** Sorbitol and ethanol production from a mixture of SM and SBH by a thermally adapted *Z. mobilis* ZM AD41 at 37 °C.

Time (h)	Treatment (SM:SBH ratio)	Sorbitol (g/L)	Ethanol (g/L)
24	FM1 (1:0)	9.80 ± 0.27^a^	35.84 ± 0.10^a^
	FM2 (3:1)	9.05 ± 0.13^b^	34.48 ± 0.25^b^
	FM3 (1:1)	4.93 ± 0.27^c^	33.46 ± 0.18^c^
	FM4 (1:3)	1.74 ± 0.13^d^	27.74 ± 0.12^d^
	FM5 (0:1)	0.45 ± 0.06^e^	20.86 ± 0.09^e^
48	FM1 (1:0)	19.24 ± 0.09^a^	36.40 ± 0.08^a^
	FM2 (3:1)	18.94 ± 0.08^b^	35.21 ± 0.09^b^
	FM3 (1:1)	6.34 ± 0.12^c^	33.83 ± 0.10^c^
	FM4 (1:3)	2.55 ± 0.08^d^	27.99 ± 0.12^d^
	FM5 (0:1)	0.56 ± 0.05^e^	21.02 ± 0.08^e^

Means ± SDs followed by different letters in the same column at each time point are significantly different at p < 0.05 based on DMRT analysis.

*Z. mobilis* is also known as an ethanologenic microorganism in addition to the conventionally used yeast *Saccharomyces cerevisiae*. This bacterium possesses several ideal properties for industrial bioethanol production, such as a high sugar uptake rate, high specific ethanol productivity and theoretical yield, and low biomass yield, making product recovery easy. As shown in the present study, Zm AD41 produced ethanol in the range of 20.86 to 35.84 g/L after 24 h and 21.02 to 36.40 g/L at 48 h after fermentation. The highest ethanol concentration was detected in the fermentation medium containing SM alone (FM1 medium), followed by a medium containing SM and SBH at a ratio of 3:1 (FM2 medium), while the lowest value was found in the fermentation medium comprising only SBH (FM5 medium). Similar to sorbitol production, SBH alone yielded the lowest value of ethanol, which may be associated with an adverse effect of a high concentration of Na on bacterial cell growth and metabolism. This finding coincided with that of Vriesekoop et al.^19^ and Chamnipa et al.^16^, who pointed out an adverse effect of high Na ions on *Z. mobilis* cell growth, morphology, and ethanol fermentation efficiency.

It can be seen in this study that fermentation media containing high portions of SBH (FM 3, 4, and 5) yielded low concentrations of sorbitol. Therefore, only FM1 and FM2 media were selected for further investigation.

### Effect of sugar concentrations on sorbitol production by *Z. mobilis* ZM AD41

Sorbitol is known as one of the byproducts formed when *Z. mobilis* is grown in a sucrose-based medium. It also forms and accumulates in a medium comprising glucose and fructose^38,39^. Previous studies demonstrated that sorbitol is primarily produced under high concentrations of sugar, specifically high sucrose concentrations, which favor the accumulation of fructose^20,40^. Since sorbitol is classified as a microbial osmoprotectant, the accumulation of sorbitol could protect microbial cells against the dehydration effects of high external osmolarity^41^. Based on this information, the effect of various sugar concentrations in the fermentation medium was evaluated in this study to test whether the production of sorbitol by a thermally adapted *Z. mobilis* ZM AD41 depends on high sugar concentration.

The results are summarized in Table 4. Increasing the sugar concentration in the fermentation medium from 150 to 200 g/L significantly enhanced sorbitol production. The maximum sorbitol concentrations produced by ZM AD41 in FM1 and FM2 media were 25.69 and 25.02 g/L, respectively, which were approximately 1.3-fold higher than those in the medium containing 150 g/L sugar. The current result agreed with a study by Barros et al.^42^, who reported a maximum sorbitol production of *Z. mobilis* in medium containing 200 g/L sucrose. Notably, a higher sugar concentration, i.e. 250 g/L, did not improve sorbitol production by thermally adapted *Z. mobilis* ZM AD41 in either FM1 or FM2 media. In contrast, a dramatic decrease in sorbitol formation was observed with an increase in the sugar concentration of the fermentation medium to 300 g/L. Sorbitol concentrations decreased to 16.16 g/L in the FM1 medium and 15.84 g/L in the FM2 medium, which was an approximately 37% reduction compared to the value detected in a medium containing 200 g/L sugar. Reducing sorbitol content at high sugar concentrations might be due to excessive osmotic pressure that negatively affects cell growth and metabolism. It is noteworthy that the results obtained in this study differed from those reported by Cazetta et al.^43^, de Barros and Celligoi^10^, and Vignoli et al.^20^, who demonstrated that a sugar concentration of 300 g/L favored the formation of sorbitol by *Z. mobilis* strain ATCC 29,191. This finding suggested that different strains of *Z. mobilis* respond to different sugar concentrations for sorbitol production.

**Table 4:** The effect of sugar concentrations on sorbitol and ethanol production by a thermally adapted *Z. mobilis* ZM AD41 at 37 °C.

Sugar concentration (g/L)	FM1		FM2	
	Sorbitol (g/L)	Ethanol (g/L)	Sorbitol (g/L)	Ethanol (g/L)
150	19.32 ± 0.06^c^	36.16 ± 0.08^c^	19.15 ± 0.08^c^	35.78 ± 0.10^c^
200	25.69 ± 0.07^a^	45.33 ± 0.08^a^	25.02 ± 0.07^a^	45.57 ± 0.06^a^
250	24.56 ± 0.08^b^	44.19 ± 0.10^b^	23.82 ± 0.09^b^	43.86 ± 0.09^b^
300	16.16 ± ± 0.09^d^	23.42 ± 0.11^d^	15.84 ± 0.10^d^	19.99 ± 0.11^d^

FM1 is a fermentation medium composed of SM and SBH at a ratio of 1:0, and FM2 is a medium composed of SM and SBH at 3:1. Means ± SDs following different letters in the same column are significantly different at p < 0.05 based on DMRT analysis.

Considering ethanol production by ZM AD41, increasing sugar concentrations in the fermentation medium also increased the ethanol content. A sugar concentration of 200 g/L yielded the highest ethanol content (45.33 g/L in FM1 medium and 45.57 g/L in FM2 medium), similar to that observed for sorbitol production. A slight reduction in ethanol content was observed when the sugar concentration in the fermentation medium increased from 200 to 250 g/L. A remarkable decrease in the ethanol concentration was detected in the medium containing 300 g/L sugar, probably due to the adverse effect of high osmotic pressure and substrate inhibition. This finding was in agreement with the studies of Sootsuwan et al.^44^ and Charoenpunthuwong et al.^45^, who reported a reduction in growth, cell viability, and ethanol fermentation efficiency of *Z. mobilis* in a medium containing a sugar concentration of 300 g/L. As demonstrated in yeast cells, high sugar levels, particularly the excess 250 g/L sugar, cause a reduction in substrate conversion rate, reducing specific ethanol productivity and yield^46,47^.

### Effect of metal ions on sorbitol production by *Z. mobilis* ZM AD41

Generally, *Z. mobilis* produced sorbitol in combination with ethanol, specifically under high sugar fermentation conditions. Several strategies have been evaluated to enhance sorbitol yield by minimizing ethanol production in *Z. mobilis*, such as the application of a cell permeabilization system^48^, high osmotic pressure conditions using concentrated fructose and glucose syrup^49^, a recombinant strain of *Z. mobilis* overexpressing the glucose-fructose oxidoreductase (*glo*) gene, and the application of divalent metal ions that inhibit enzymes involved in the ethanol production pathway^12^. Applying divalent metal ions seemed to be a promising and cost-effective method for improving sorbitol yield. In this study, various metal ions, including ZnSO_4_.7H_2_O, CaCl_2_, FeSO_4_.7H_2_O, MgSO_4_.7H_2_O, MnSO_4_.H_2_O, and CuSO_4_.5H_2_O, at the concentrations selected based on a study of Liu et al.^12^ were tested for their effect on sorbitol production by ZM AD41. FM1 and FM2 media containing SM and SBH at ratios of 1:0 and 3:1, respectively, with a sugar concentration of 200 g/L, were used in this experiment. The results are summarized in Table 5.

**Table 5:** Effect of metal ions on sorbitol and ethanol production by a thermally adapted *Z. mobilis* ZM AD41 at 37 °C.

Treatment	Metal ion	Concentration (g/L)	FM1		FM2	
			Sorbitol (g/L)	Ethanol (g/L)	Sorbitol (g/L)	Ethanol (g/L)
1	Control	0.0	25.13 ± 0.10^cd^	45.13 ± 0.09^a^	25.09 ± 0.08^c^	45.36 ± 0.10^a^
2	ZnSO_4_.7H_2_O	1.0	27.46 ± 0.11^b^	35.36 ± 0.09^d^	26.87 ± 0.08^b^	34.94 ± 0.07^d^
3	CaCl_2_	5.0	30.31 ± 0.10^a^	31.94 ± 0.08^f^	30.01 ± 0.07^a^	32.09 ± 0.09^g^
4	FeSO_4_.7H_2_O	2.0	24.96 ± 0.11^de^	35.95 ± 0.07^c^	25.11 ± 0.08^c^	35.46 ± 0.13^c^
5	MgSO_4_.7H_2_O	2.5	25.25 ± 0.11^c^	36.75 ± 0.06^b^	25.16 ± 0.07^c^	36.33 ± 0.06^b^
6	MnSO_4_.H_2_O	2.0	25.06 ± 0.10^de^	35.01 ± 0.08^e^	24.53 ± 0.09^d^	34.52 ± 0.09^e^
7	CuSO_4_.5H_2_O	0.5	24.94 ± 0.07^e^	35.04 ± 0.09^e^	24.45 ± 0.12^d^	34.17 ± 0.11^f^

Means ± SDs following different letters in the same column are significantly different at p < 0.05 based on DMRT analysis.

As shown in Table 5, the addition of CaCl_2_ into the FM1 medium significantly enhanced the sorbitol production of ZM AD41 by 20.6% and effectively reduced the ethanol formation by approximately 29.23% compared to the control treatment where no metal ion was added. For the FM2 medium, CaCl_2_ addition also significantly increased sorbitol production by approximately 19.6% and markedly reduced ethanol production by 29.26% compared to the control treatment. In addition, supplementation of the fermentation medium with ZnSO_4_.7H_2_O slightly promoted sorbitol production of ZM AD41 by 9.3% in the FM1 medium and approximately 7.0% in the FM2 medium compared to the control treatment without metal ion supplementation. The ethanol concentrations in the fermentation medium supplemented with ZnSO_4_.7H_2_O also reduced by approximately 21.6% and 23.0% in the FM1 and FM2 media, respectively. These findings suggested that CaCl_2_ and ZnSO_4_.7H_2_O could alter the main production pathway from ethanol to sorbitol by inactivating or inhibiting the enzymes associated with the ethanol production pathway in *Z. mobilis*, as previously demonstrated by Liu et al.^12^ and Chamnipa et al.^16^.

It should be noted in the current study that the addition of other divalent metal ions did not promote sorbitol production, even though these metal ions could reduce ethanol formation by ZM AD41. In addition, a slight decrease in sorbitol production efficiency was observed when FeSO_4_.7H_2_O and CuSO_4_.5H_2_O were added to the FM1 medium or when MnSO_4_.H_2_O and CuSO_5_.5H_2_O were added to the FM2 medium. These observations differed from those reported by Liu et al.^12^, who noted that supplementation with Fe^2+^, Mg^2+^, Mn^2+^, and Cu^2+^ significantly enhanced the sorbitol yield from a mixture of glucose-fructose medium by recombinant *Z. mobilis* overexpressing the *glo* gene. The difference in the results between this study and that of Liu et al.^12^ might be due to the differences in bacterial strain and fermentation medium. SM and SBH comprise several metal ions compared to glucose and fructose. Excessive addition of Fe^2+^, Cu^2+^, and Mn^2+^ may cause a negative effect on bacterial growth and metabolism, leading to a reduction of both sorbitol and ethanol yield.

**Figure 2:**
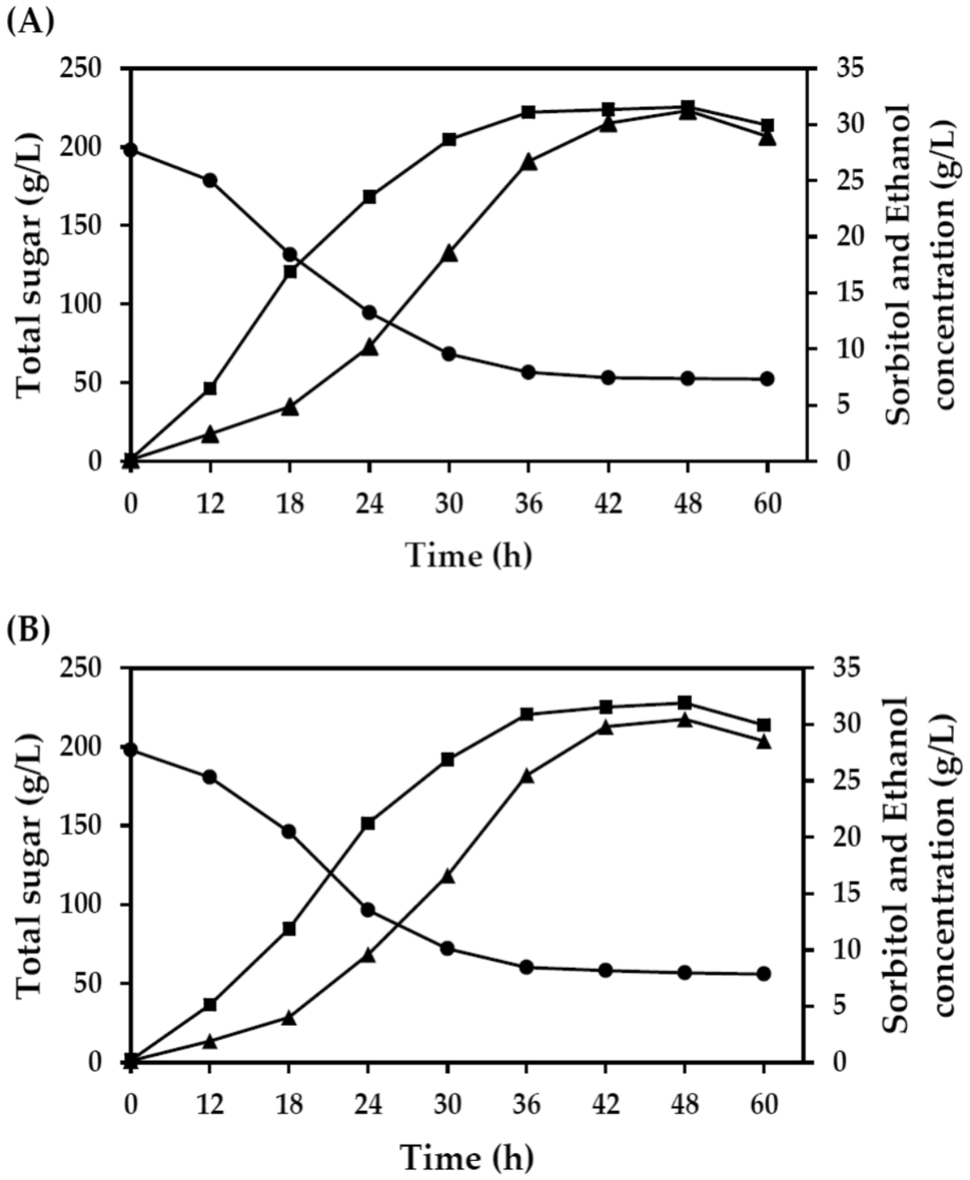
Sorbitol and ethanol production by a thermally adapted *Z. mobilis* ZM AD41 in a 2-L Erlenmeyer flask at 37 °C using FM1 medium containing SM (**A**) and FM2 medium containing a mixture of SM and SBH (**B**). (Black color filled circle), total sugar; (Black color filled square), ethanol; (Black color filled triangle), sorbitol.

Based on the results in Table 5, CaCl_2_ was chosen, and sorbitol production in a 2-L Erlenmeyer flask under semi-anaerobic conditions by statically incubated at 37 °C was investigated. As shown in Fig. 2, sorbitol was gradually produced during the first 12 h after fermentation. Its production markedly increased after that, reaching a maximum of 31.23 g/L in FM1 medium and 30.45 g/L in FM2 medium at 48 h after fermentation, with productivity values of 0.65 g/L.h and 0.63 g/L.h, respectively. The production of sorbitol slightly decreased after 48 h of fermentation, possibly due to the depletion of carbon and nutrients in the fermentation medium. Another possibility is that sorbitol may be used as a carbon and energy source for bacterial growth and metabolism, similar to those reported in the literature^16,50,51^.

*Z. mobilis* ZM AD41 also produced relatively high ethanol concentrations when cultivated in a 2-L Erlenmeyer flask. The ethanol production profile was similar to that of sorbitol production. The maximum ethanol concentrations produced by ZM AD41 in the FM1 and FM2 media were 31.56 g/L and 31.89 g/L, respectively, almost similar to those observed in the experiment carried out in a 250-mL Erlenmeyer flask. These findings indicated that the fermentation conditions established in the current study could promote sorbitol production efficacy by minimizing the ethanol production of a thermally adapted *Z. mobilis* ZM AD41 using SM and SBH as feedstock.

A comparative analysis of sorbitol production using different feedstock and strains of *Z. mobilis* was performed, and the results are summarized in Table 6. The maximum sorbitol concentration produced by a thermally adapted *Z. mobilis* ZM AD41 using SM (31.23 g/L) or a mixture of SM and SBH (30.45 g/L) at a sugar concentration of 200 g/L was greater than that of Chamnipa et al.^16^, who reported a maximum sorbitol content of 5.89 g/L by *Z. mobilis* TISTR548 using a mixture of SBH and cassava pulp hydrolysate (CPH) as feedstock. In addition, the sorbitol content in the current study was also comparable to those of Vignoli et al.^11^ and Vignoli et al.^20^, using sucrose or a mixture of sucrose and maltose as feedstock. Notably, a high sorbitol concentration (161.10 g/L) was also recorded by recombinant *Z. mobilis* overexpressing the *glo* gene using a mixture of glucose and fructose as raw material at a sugar concentration of 300 g/L^12^. Another study by de Barros et al.^10^ using *Z. mobilis* ATCC29191 also showed high sorbitol production (60.42 g/L) when 300 g/L of invertase-treated sucrose was used as feedstock. Thus, further studies should be performed to enhance sorbitol production efficiency, such as overexpression of the *glo* gene in the thermally adapted *Z. mobilis* ZM AD41 using a genetic engineering approach or optimization conditions for sorbitol production using a statistical experimental model or application of a cell permeabilization system.

**Table 6:** Sorbitol production by different strains of *Z. mobilis* using different feedstock.

Strains	Feedstock	Feedstock concentration (g/L)	Sorbitol (g/L)	References
*Z. mobilis* ATCC29191	Sucrose	300	32.34	^11^
*Z. mobilis* ATCC29191	Sucrose	300	38.68	^20^
	Sucrose (200 g/L) and maltose (100 g/L)	300	38.07	
*Z. mobilis* ATCC29191	Sucrose (treated with yeast invertase)	300	60.42	^10^
*Z. mobilis* ATCC29191	Sucrose	200	36.09	^42^
*Z. mobilis* TISTR548	SBH and CPH mixture	140	5.89	^16^
*Z. mobilis* ZM4 (pHW20a-*gfo*)	Glucose and fructose mixture	300	161.10	^12^
*Z. mobilis* ZM AD41	SM	200	31.23	This study
	SM and SBH mixture	200	30.45	This study

*SM* sugarcane molasses, *SBH* sugarcane bagasse hydrolysate, *CPH* cassava pulp hydrolysate.

## Conclusion

As demonstrated in the present study, the thermally adapted *Z. mobilis* ZM AD41 exhibited a high potential in producing sorbitol from SM or a mixture of SM and SBH. The fermentation medium containing SM or a mixture of SM and SBH at a ratio of 3:1 (FM2 medium) as a sole carbon source was the best substrate for sorbitol production at 37 °C by ZM AD41. The optimum sugar concentration in a fermentation medium was 200 g/L, while supplementation of 5.0 g/L CaCl_2_ into a fermentation medium significantly enhanced sorbitol production by ZM AD41. Furthermore, adding CaCl_2_ into a fermentation medium significantly reduced the ethanol formation of thermally adapted *Z. mobilis* ZM AD41. The maximum sorbitol content produced by ZM AD41 in a 2-L Erlenmeyer flask using the fermentation medium containing SM alone or a mixture of SM and SBH at 3:1 was 31.23 g/L and 30.45 g/L, corresponding to productivity of 0.65 g/L.h and 0.63 g/L.h, respectively. This finding demonstrated that byproducts from the sugarcane manufacturing process, specifically SB, which is considered a lignocellulosic material, could be used as an alternative substrate for sustainable sorbitol production by supplementing or blending its undetoxified hydrolysate with SM at the optimum concentration. The results in this study also pointed out that a thermally adapted *Z. mobilis* ZM AD41 has a high potential for sorbitol production at a relatively high-temperature fermentation condition using agricultural wastes, particularly SM or SBH, as feedstock. Therefore, the operation cost of the production process could be reduced since the energy required for a cooling system can be minimized.

## Data Availability

All data generated or analyzed during this study are included in this published article.
